# Monitoring Atrial Fibrillation Using Wearable Digital Technologies: The Emerging Role of Smartwatches

**DOI:** 10.3390/jcm15010014

**Published:** 2025-12-19

**Authors:** Panagiotis Stachteas, Marios G. Bantidos, Nikolaos Papoutsidakis, Athina Nasoufidou, Paschalis Karakasis, Georgios Sidiropoulos, Christos Kofos, Dimitrios Patoulias, Vasileios Ediaroglou, George Stavropoulos, Efstratios Karagiannidis, Barbara Fyntanidou, Dimitrios Tsalikakis, Emmanouil Smyrnakis, George Kassimis, Christodoulos E. Papadopoulos, Nikolaos Fragakis

**Affiliations:** 1 Second Department of Cardiology, Hippokration General Hospital of Thessaloniki, Aristotle University of Thessaloniki, 54642 Thessaloniki, Greece; mbadidos@gmail.com (M.G.B.); nipapou4@gmail.com (N.P.); athinanassi@gmail.com (A.N.); pakar15@hotmail.com (P.K.); sidiropoulos.georges@gmail.com (G.S.); chriskofos21@gmail.com (C.K.); stavropoulosgeo@gmail.com (G.S.); gksup@yahoo.gr (G.K.); nfrag@auth.gr (N.F.); 2 Second Propedeutic Department of Internal Medicine, Hippokration General Hospital of Thessaloniki, Aristotle University of Thessaloniki, 54642 Thessaloniki, Greece; dipatoulias@gmail.com; 3 Department of Emergency Medicine, AHEPA University Hospital, Aristotle University of Thessaloniki, 54636 Thessaloniki, Greece; billedi03@gmail.com (V.E.); stratoskarag@gmail.com (E.K.); bfyntan@yahoo.com (B.F.); 4 Department of Electrical and Computer Engineering, University of Western Macedonia, 50100 Kozani, Greece; tsalikakis@gmail.com; 5 Laboratory of Primary Health Care, General Practice and Health Services Research-Medical School, Aristotle University of Thessaloniki, 54124 Thessaloniki, Greece; smyrnak@auth.gr; 6 Third Cardiology Department, Medical School, Hippokration General Hospital of Thessaloniki, Aristotle University of Thessaloniki, 54642 Thessaloniki, Greece; chrpapado@gmail.com

**Keywords:** artificial intelligence, atrial fibrillation, digital health, monitoring, single-lead electrocardiography, smartwatch, wearables

## Abstract

Atrial fibrillation (AF) is the most common sustained arrhythmia and a growing global health burden, yet conventional monitoring with Holter devices, event recorders and implantable loop recorders often fails to adequately capture recurrence. Rapid advances in digital health, wearable biosensors and artificial intelligence (AI) have transformed consumer smartwatches and wearables into potential clinical tools capable of continuous, real-world rhythm surveillance. This narrative review synthesizes contemporary evidence on smartwatch-based AF monitoring, spanning core technologies—photoplethysmography, single-lead electrocardiography and AI fusion algorithms—and validation studies across post-ablation follow-up. Compared with traditional modalities, smartwatch-based AF monitoring demonstrates improved detection of AF recurrence, enhanced characterization of AF burden, symptom–rhythm correlation, and greater patient engagement. At the same time, key limitations are critically examined, including motion artifacts, false-positive alerts, short recording windows, adherence dependence, digital literacy and access gaps, as well as unresolved issues around regulation, interoperability and data privacy. By integrating engineering advances with guideline-directed care pathways, smartwatch-based AF monitoring holds promise to complement, rather than immediately replace, established diagnostic tools and to enable more proactive, individualized AF management. Future work must focus on robust clinical validation, equitable implementation and clear regulatory frameworks to safely scale these technologies.

## 1. Introduction

Atrial fibrillation (AF) represents the most prevalent sustained cardiac arrhythmia worldwide, affecting an estimated 37.5 million individuals globally, with projections indicating a more than 60% increase by 2050 [1]. This rising burden is driven primarily by population aging, increasing comorbidity prevalence, and enhanced detection capabilities [1,2]. AF is associated with profound clinical consequences, including a five-fold increased risk of ischemic stroke, elevated heart failure (HF) incidence, cognitive decline, cardiovascular morbidity and all-cause mortality [1,3]. The socioeconomic impact is equally substantial as direct healthcare costs attributable to AF range from €2315 to €3307 per patient annually in Europe and $6410 to $14,083 in the United States, accounting for approximately 1% of national healthcare expenditures [4,5,6]. These figures underscore AF as a critical public health challenge demanding innovative approaches to early detection, risk stratification, and personalized management [2].

Traditional AF monitoring modalities—including patient-reported symptom questionnaires, 24–72 h Holter monitoring, extended external event recorders, and implantable loop recorders (ILRs)—have served as the cornerstone of arrhythmia surveillance for decades. However, these conventional approaches are constrained by limited monitoring duration, patient discomfort, reduced adherence, and insufficient sensitivity for detecting paroxysmal or asymptomatic AF episodes that occur unpredictably between clinical encounters [7,8]. Furthermore, the episodic nature of traditional monitoring hinders accurate AF burden quantification, a parameter increasingly recognized as a critical determinant of stroke risk and therapeutic decision-making [9,10].

The convergence of digital health technologies, wearable biosensors, and artificial intelligence (AI)-driven algorithms has catalyzed a paradigm shift in cardiovascular monitoring, enabling continuous, patient-centered, and real-world cardiac rhythm assessment outside traditional healthcare settings [11,12]. Modern consumer-grade and medical-grade wearables—including smartwatches, sensor patches, and smartphone-paired electrocardiographic devices—employ photoplethysmography (PPG) and single-lead electrocardiography (ECG) to passively detect irregular heart rhythms with high diagnostic accuracy [13,14]. Landmark studies such as the Apple Heart Study, Huawei Heart Study, and eBRAVE-AF trial have demonstrated the feasibility, scalability, and clinical impact of wearable-based AF screening, with detection rates more than doubling compared to usual care and facilitating timely initiation of oral anticoagulation therapy [15,16].

The integration of AI-enhanced algorithms into wearable platforms has further amplified their diagnostic precision, enabling real-time arrhythmia classification, motion artifact filtering, and longitudinal AF burden estimation [17,18]. These innovations support a transformative shift from reactive, hospital-based episodic care to proactive, continuous, and participatory cardiovascular health management [19]. Importantly, these technological advances intersect with evolving insights into atrial substrate (e.g., atrial cardiomyopathy and fibrosis), reinforcing the concept that rhythm phenotype (burden, temporal patterns) and atrial substrate together determine prognosis and management choices [20,21]. As digital health ecosystems mature and regulatory frameworks evolve, the role of wearables in AF care extends beyond screening to encompass therapeutic guidance, post-ablation monitoring, and personalized risk stratification.

Despite their promise, the widespread clinical adoption of smartwatch-based AF monitoring faces persistent challenges, including algorithmic reliability concerns, false-positive alerts, regulatory heterogeneity, data privacy issues, and digital health disparities among vulnerable populations [22,23,24]. Addressing these barriers through robust validation studies, standardized evaluation frameworks, equitable access initiatives, and international regulatory cooperation is essential to realize the full potential of wearable technologies in improving AF detection, management, and outcomes at scale. This narrative review provides a comprehensive examination of the current state and future directions of smartwatch-based AF monitoring, synthesizing evidence on core technologies, clinical advantages, limitations, and integration strategies to guide clinicians, researchers, and policymakers in this rapidly evolving field.

## 2. Methods

A narrative literature review of the existing evidence was undertaken to synthesize and critically appraise the heterogeneous data on the use of wearable digital technologies in AF monitoring. The PubMed/MEDLINE, Scopus, Web of Science, and Google Scholar databases were systematically searched from inception up to November 2025 using Boolean operators (AND, OR, NOT) with appropriate combinations of the following MeSH terms: “atrial fibrillation”, “wearable devices”, “smartwatch”, “photoplethysmography”, “single-lead electrocardiography”, “artificial intelligence”, “remote monitoring” and “arrhythmia detection”. Reference lists of eligible studies were also screened to identify additional relevant publications. Only original research, systematic reviews, meta-analyses, and clinical trials published in peer-reviewed journals written in English were included, while gray literature, conference abstracts, and non-peer-reviewed sources were excluded. From the initial pool of retrieved records, duplicates were removed, and a first-level screening was performed based on titles and abstracts by three independent investigators (PS, MGB, NP). Full-text articles were subsequently assessed for eligibility, and studies focus on wearable technologies (smartwatches, wristbands, smartphone-based devices) for AF detection or monitoring were included. Disagreements were resolved through consensus discussion.

## 3. Traditional Methods for AF Monitoring

A variety of established modalities are currently available for AF monitoring, each offering distinct advantages and inherent limitations depending on the clinical scenario. Among the most accessible approaches are patient-reported outcome measures, which aim to quantify symptom burden and the perceived impact of AF on quality of life. One such tool is the Atrial Fibrillation Effect on QualiTy of Life (AFEQT) questionnaire, a validated and AF-specific instrument comprising 20 items that assess symptom severity, limitations in daily activities, treatment-related concerns, and treatment satisfaction [25]. Despite its clinical utility and widespread adoption, this patient-driven assessment is subject to inherent variability, as symptom perception and AF-related experiences differ substantially among individuals, potentially limiting its objectivity and comparability across the patient population [26].

Ambulatory electrocardiographic monitoring using 24–72 h Holter devices provides a more objective modality for AF surveillance. Holter monitoring facilitates characterization of AF subtype (paroxysmal, persistent, or permanent) and allows assessment of therapeutic response, particularly with respect to ventricular rate control. Additionally, it may aid in identifying potential arrhythmogenic triggers contributing to AF onset [7]. Historically, Holter monitors represented the primary option for extended rhythm assessment. However, their clinical use has declined in recent years due to the discomfort associated with device size and bulk, which may reduce patient adherence [7,8]. Furthermore, the relatively short duration of monitoring limits the sensitivity for detecting infrequent or paroxysmal AF episodes, contributing to underdiagnosis in certain populations [27].

Compared with conventional Holter monitoring, extended external event recorders may offer superior diagnostic yield for detecting AF episodes, particularly those occurring infrequently [28]. These non-invasive devices can typically be employed for up to 30 days and are activated either by the patient in response to symptoms or automatically through embedded arrhythmia-detection algorithms. Upon activation, they capture a predefined interval of electrocardiographic data both preceding and following the trigger event. Despite the advantage of prolonged monitoring, this modality may fail to document the full duration of AF episodes and remains limited in identifying asymptomatic arrhythmias that do not meet activation criteria. Additionally, prolonged use of surface electrodes can cause patient discomfort or skin irritation, potentially impacting adherence and data quality [29].

Finally, implantable loop recorders (ILRs) represent a highly valuable modality for long-term AF monitoring. These devices are inserted subcutaneously through a minimally invasive procedure and provide continuous rhythm surveillance with high sensitivity for AF detection. However, specificity may be reduced due to false-positive recordings, often related to oversensing of myopotentials or other non-arrhythmic electrical signals [30]. ILRs enable extended monitoring for up to three years without requiring patient activation or adherence to external equipment. Despite these advantages, their invasive nature and comparatively high cost remain important considerations that may limit their broader implementation in certain clinical contexts [31].

## 4. Core Technologies and Commercial Applications in AF Wearables

**Figure 1:**
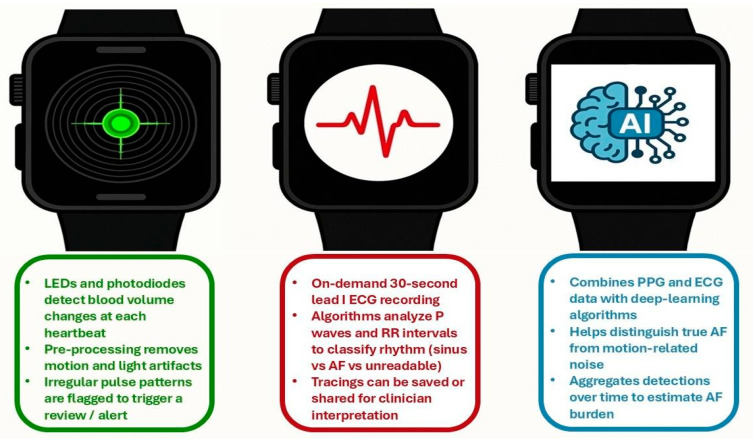
Core smartwatch-based technologies for atrial fibrillation (AF) monitoring. The left panel illustrates photoplethysmography (PPG), the middle panel depicts single-lead ECG recording, and the right panel represents artificial intelligence (AI)-based fusion algorithms integrating smartwatch signals for AF detection.

In recent years, remarkable progress has been made regarding consumer-grade wearables, paving the way from simple fitness trackers into potential clinically implemented cardiac monitors. Future applications, expanding from first AF detection to continuous rhythm surveillance and burden quantification, would depend on three core technological modalities (Figure 1). PPG leverages green and infrared light emitting diodes (LEDs) paired with photodiodes to detect volumetric changes in peripheral blood vessels at each cardiac cycle [13,14]. By sampling at 25–100 Hz, modern smartwatches capture beat-to-beat pulse intervals and waveform morphology. To ensure signal quality, advanced preprocessing pipelines remove motion artifacts and ambient-light interference, enabling the extraction of features such as pulse-interval variability, amplitude ratios, and morphological metrics. Using statistical models, such as return maps/Poincare Plots, episodes that exceed predefined thresholds are marked as irregular, automatically triggering review or alerting the user [32].

Single-lead ECG hardware (typically one electrode on the case back and a second on the crown or bezel) gives the wearer a 30 s lead I tracing on demand, recorded at 250–500 Hz and 0.5–40 Hz band-pass filtered (frequencies outside the range are rejected) [33,34,35]. QRS-detection and P-wave analysis algorithms measure RR intervals, PR intervals, and waveform morphology to classify rhythms as sinus, AF, or “unreadable” [36]. Tracings can be stored locally on the paired smartphone, exported in standardized formats, such as PDF files, and/or uploaded to cloud-based platforms for asynchronous clinician interpretation or further automated rhythm classification. Finally, device-embedded fusion classifiers integrate time-series features from both PPG and ECG sensors via deep-learning architectures [e.g., convolutional neural networks (CNNs) or transformers] [37]. This method improves discrimination between true AF and motion-induced artifacts (reports of false positives reduction by up to 60%) and estimation of AF burden over extended periods, aggregating probabilistic outputs across minutes to days [11,12].

The translation of these technologies into commercially viable products has fueled rapid growth in the market of wearable health innovation, including leading technology companies. The latest Apple Watch Series integrates PPG, single-lead ECG and an AF history feature that estimates the proportion of time spent in AF, automatically uploading flagged episodes to the Health application dashboard [38,39]. The low AF notification rate in the Apple Heart Study reflects intentional high specificity to minimize false positives and alert fatigue, not algorithm failure. This design reduces sensitivity, so some true AF episodes are missed, consistent with the intermittent nature of paroxysmal AF. The 84% PPV indicates AF was present at the time of alert; absence on later ECG monitoring reflects natural AF variability, not misclassification. An underemphasized finding is that notified participants had a median AF burden of 7%, higher than the ~2% seen in asymptomatic AF detected by implantable devices. Because AF burden >5% is linked to higher stroke risk, the study likely identified a higher-risk subgroup. Key limitations include potential overdiagnosis of minimal-burden AF and uncertain net benefit of anticoagulation given bleeding risk. CHA_2_DS_2_-VASc was not validated in wearable-detected silent AF and is insufficient alone. Anticoagulation decisions should integrate AF burden, bleeding risk, and patient preferences, emphasizing shared decision-making [38,39].

Fitbit devices, including the Sense, Versa (2–4) and Charge (3–5) series, incorporate an FDA-cleared, PPG-based irregular heart rhythm notification (IHRN) algorithm that opportunistically analyses 5 min pulse-wave tachograms (pulse windows) and alerts the user to irregularities [40]. The Samsung Galaxy Watch line pairs on-demand single-lead ECG recording with a passive, PPG-based IHRN service within the Samsung Health Monitor platform [41]. Lastly, the AliveCor KardiaMobile is a handheld (two-finger configuration), smartphone-paired device, FDA-approved for AF detection, that remains among the most extensively published direct-to-consumer gadgets in AF validation research [39,41].

## 5. Accuracy and Diagnostic Performance of AF Wearables

Wearable digital technologies seem to be a promising alternative method in detecting AF recurrence in patients with paroxysmal AF. The primary focus of the study was to compare the effectiveness of this innovation with established monitoring modalities, including ILRs and Holter monitors. Particular attention was also given to the detection of asymptomatic and paroxysmal AF. A concise summary of the current evidence is presented in Table 1.

Post-ablation monitoring was conducted using both conventional Holter devices and wearable digital technologies in two studies. The application of wearable systems was associated with enhanced detection rates of AF recurrence compared to standard Holter monitoring methods [43,46]. Specifically, in one study employing the AliveCor KardiaMobile device, patients performed scheduled heart rhythm recordings three times daily and during symptomatic episodes for 30 s. This approach resulted in the identification of 29 AF recurrences (sensitivity: 95.3%, specificity: 97.5%, PPV = 76.5%, NPV = 99.6%), whereas conventional Holter monitoring detected only 17 episodes. Consistently, Sikorska et al. [46] found that daily electrocardiographic transmissions via wearable devices registered 12 episodes, while Holter monitors identified 7 AF recurrences. In addition, wearable technologies were generally well-tolerated among patients and were linked to earlier detection of AF recurrence. Further investigations produced similar outcomes, demonstrating more rapid identification of AF episodes in the postoperative period when wearable devices were utilized [42,48,49].

Comparative analysis of ILRs and consumer-grade wrist-worn PPG devices revealed distinct performance characteristics for AF recurrence detection. PPG-based wrist devices exhibited high sensitivity but comparatively low specificity, necessitating confirmatory ECG verification due to the modest positive predictive value (PPV) [50]. The protocols for wearable monitoring required patients to submit 60 s ECG recordings three times daily and during symptomatic episodes, with comprehensive documentation of symptoms including palpitations, chest pain, dyspnea, confusion, and fatigue. The use of wearable technology enabled direct mapping of symptom–rhythm correlation (SRC) and underscored the clinical importance of evaluating AF burden—the proportion of time spent in AF—rather than simply noting arrhythmia presence or absence [51]. This approach facilitated nuanced characterization of AF recurrences as paroxysmal or persistent, based on correlated clinical and rhythm findings. Wearable devices demonstrated a patient-centered approach, actively promoting patient engagement in disease management, which aligns with contemporary AF management guidelines [43,53]. Notably, patients who experienced episodes of atrial tachyarrhythmia displayed enhanced adherence and motivation for sustained, continuous monitoring using these wearable platforms [48].

Nevertheless, this feature may also present challenges, as the success of follow-up strategies depends heavily on the patient’s commitment and compliance in maintaining regular rhythm recordings. As observed in one of our studies, home-based ECG recordings at least one time per day using the Complete device (ECG paired with a blood pressure monitor; Omron Healthcare, Kobe University Hospital, Kobe, Japan) identified a greater number of AF recurrences than Holter monitoring within a shorter observation period; however, the effectiveness of post-treatment monitoring was notably adherence-dependent [47]. Even though patients were trained during their hospitalization on how to use the device and help was provided in person or through the call-center during the whole monitoring period, approximately one quarter of participants discontinued follow-up. Several limitations have also been associated with the use of wearable technologies for detecting AF recurrence. First, operational difficulties and user-related issues led to the exclusion of certain participants, as no valid recordings were obtained [43]. The same study also underscored the short recording duration (30 s) as a limiting factor, potentially resulting in the underestimation of AF episodes. Another critical limitation involves motion artifacts and signal noise interference, both of which can significantly affect arrhythmia detection algorithms and consequently compromise the accuracy of the recorded data [50]. Slight increase in noise was observed in another study in which a smart bra-type device equipped with the dry textile electrode (hitoe) was used [45]. The episodes of AF recurrence (monitored as continuous on the electrode ECG) were consistent with the results of Holter ECG but were fragmented by these artifacts. Nevertheless, the discrepancy in AF duration was 0.6% which was characterized as clinically insignificant.

According to another study which applied automated and AI algorithms (besides cardiologist’s view) to analyze the ECGs, the portable device used demonstrated higher percentages of AF recurrences found in a shorter period of time with 23.8% of these episodes being asymptomatic [44]. Worth mentioning is the fact that the AI algorithm elevated the accuracy of the ECG reading (Se = 94.4% and Sp = 98.5% compared to the automated detection algorithm with Se = 90.7% and Sp = 96.2%). Also, the patients in the group monitored by the handheld single-lead ECG monitor were more adherent to the oral anticoagulation given. Recent studies have underscored the utility of consumer-grade smartwatches in the detection of AF. In particular, algorithms implemented in the Apple Watch and Fitbit platforms demonstrated a strong correlation with implantable cardiac monitor–derived AF burden (Pearson r > 0.97 for all evaluated algorithms), with detection sensitivities reaching 82% [52]. Recordings were obtained from post-ablation patients during presumed daytime hours (08:00 AM–10:00 PM), and AF burden in undetected episodes was minimal (median < 0.02%). Finally, a recently published study reported high accuracy, sensitivity, and specificity of smartwatch-based AF identification; however, it emphasized the need for further validation using real-world data [54].

However, several methodological limitations warrant consideration. Many of the available studies enrolled relatively small cohorts, which limits statistical power and the precision of diagnostic accuracy estimates [42,44,45,46,50]. In addition, a number of studies were conducted in single centers, raising concerns about center-specific practice patterns and referral biases [42,48]. Selection bias is also likely in Hermans et al. study, as only patients willing to use the provided handheld ECG device (AliveCor KardiaMobile) were recruited, while Sandgren et al. enrolled post-ablation patients who consented to install and use a smartphone application [43,51]. Such inclusion criteria favor digitally engaged, relatively health-literate individuals and may under-represent older, frailer or socioeconomically disadvantaged patients with AF, so caution is required when extrapolating these findings to the broader AF population. Finally, short follow-up duration in several studies constrains assessment of long-term AF recurrence and downstream clinical outcomes [42,44,46].

## 6. Advantages of Smartwatch-Based AF Monitoring

Smartwatch technologies have emerged as transformative tools in AF detection and management, offering substantial advantages over conventional monitoring approaches [16]. These devices employ high-frequency PPG sampling and on-demand single-lead ECG algorithms to provide continuous, real-world cardiac rhythm surveillance that extends beyond traditional intermittent in-clinic assessments. The capacity for extended, passive data acquisition represents a major clinical advantage, improving detection of paroxysmal and asymptomatic AF episodes, while permitting estimation of AF burden across extended time periods [16,55]. Unlike conventional Holter monitors that capture 24–48 h of data, smartwatches enable continuous rhythm monitoring over weeks to months during routine daily activities. Recent evidence demonstrates excellent correlation between smartwatch-derived AF burden estimates and reference Holter monitoring standards, with intraclass correlation coefficients exceeding 0.92 for integrated algorithms [56]. Approximately 68% of users maintain engagement at one year, enabling sustained long-term monitoring in real-world populations [57]. The passive nature of PPG-based detection minimizes patient burden while maximizing capture of paroxysmal AF episodes that occur unpredictably between clinical visits. These capabilities are increasingly relevant as emerging evidence links higher AF burden to elevated stroke and HF risk, underscoring the clinical utility of dense longitudinal data streams [58].

In parallel, smartwatch technology fundamentally shifts AF management paradigm by enabling patients to actively participate in their health monitoring. Evidence demonstrates that AF patients are significantly more likely to share wearable-derived health data with healthcare providers, reflecting heightened disease awareness and engagement [59,60]. Real-time device alerts (e.g., automated irregular rhythm notifications), enable prompt symptom recognition and appropriate medical consultation, supporting earlier presentation and evaluation [59,60,61]. Integration with mobile health applications and structured remote monitoring programs facilitates enhanced patient-provider communication and supports medication adherence, optimization of therapy and lifestyle modifications [59,61]. The accessibility of these devices extends cardiac monitoring to populations facing traditional healthcare barriers, thereby democratizing access to advanced cardiovascular surveillance.

Remote monitoring capabilities enable early AF detection with significant stroke prevention implications. The randomized eBRAVE-AF trial demonstrated that smartphone-based PPG screening more than doubled treatment-relevant AF detection rates compared to usual care (1.33% vs. 0.63%, OR 2.12), with subsequent oral anticoagulation initiation in newly diagnosed patients [62]. Cost-effectiveness analyses indicate that wearable screening reduces stroke incidence by 20–23 cases per 100,000 person-years with an incremental cost-effectiveness ratio of $57,894 per quality-adjusted life year—within accepted thresholds [63]. Early detection has prevented progression to complications, as illustrated by cases of arrhythmia-induced cardiomyopathy successfully managed following smartwatch alerts [64].

Although major professional societies emphasize that smartwatch detections must be interpreted within a validated clinical framework—including confirmatory ECG and CHA_2_DS_2_-VASc-guided assessment—the technology offers a promising adjunct to guide patient-specific decision-making and monitor therapeutic response [52,65]. The Up to AF trial represents pioneering work in smartwatch-guided anticoagulation therapy, where direct oral anticoagulant dosing is individualized based on real-time Apple Watch AF detection, potentially reducing unnecessary anticoagulation exposure while maintaining stroke protection [66]. Post-ablation monitoring demonstrates particular utility, with smartwatch algorithms achieving 82.2% sensitivity for AF recurrence detection and correlation exceeding 0.97 with implantable cardiac monitor data—superior to conventional intermittent monitoring [66]. Wearable devices facilitate remote heart rate monitoring during rate control therapy optimization, capturing extensive data that inform therapeutic adjustments [65]. Integration of wearable data into clinical workflows represents a fundamental advancement in personalized, responsive AF management that bridges in-clinic assessments and continuous real-world monitoring.

## 7. Challenges and Limitations of Smartwatch-Based AF Monitoring

**Figure 2:**
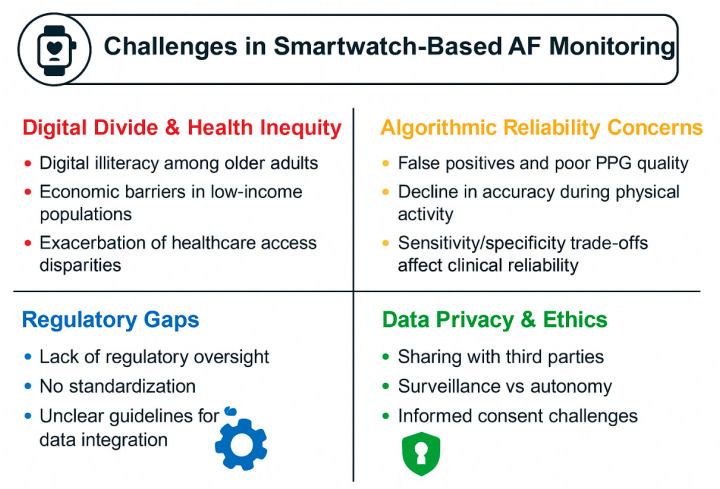
Main challenges associated with atrial fibrillation (AF) monitoring using wearable smartwatches.

Monitoring AF using wearable smartwatches introduces significant challenges that should be critically examined when evaluating their clinical adoption (Figure 2). One major barrier is digital illiteracy among vulnerable populations including older adults-including people with frailty or dementia- and individuals from lower socioeconomic backgrounds [22,23,24]. Studies show that the median age of AF patients aligns with populations that often lack familiarity with smartphone and smartwatch interfaces, leading to reduced engagement and potential misuse of these devices [67]. Digital literacy disparities correlate with social determinants of health, where low-income groups—including refugees and immigrants and socially vulnerable populations in general—may have limited access to the necessary devices or internet connectivity, compounding health inequities [68,69,70,71]. Limited access to appropriate technological devices, such as smartwatches, and reliable internet connectivity significantly hinders the use of wearable-based AF monitoring through telemedicine in remote, island, and low-income regions [72]. These barriers prevent effective real-time AF detection and management, disproportionately affecting populations that could benefit most from early arrhythmia identification and intervention via smart health technologies [73]. Educational initiatives and user-centric design principles emphasizing simplicity, large icons, and guided interfaces are essential to broaden access [74,75]. Otherwise, the benefits of early AF detection risk remaining confined to younger, technologically adept subsets of the population.

Algorithmic reliability in smartwatch-based AF detection remains a significant constraint to clinical adoption. A primary concern is the occurrence of false-positive detections, predominantly attributable to motion artifact interference and suboptimal PPG signal quality, which frequently precipitate unnecessary diagnostic evaluation, heightened patient anxiety, and potential healthcare resource overutilization [14,32,76]. Arrhythmias other than AF—particularly premature atrial contractions (PACs) and premature ventricular contractions (PVCs)—constitute major sources of false-positive alerts due to their resemblance to AF-induced heart rate irregularity patterns on PPG analysis [77,78]. Studies employing machine learning approaches initially trained on limited, imbalanced datasets reported that several false-positive detections occurred in cohorts with frequent PACs, whereas no false positives emerged in patients with stable sinus rhythm, underscoring the specificity-reducing impact of premature ectopy [78]. The deterioration of smartwatch algorithm performance during physically demanding conditions represents an additional practical limitation; continuous PPG-based monitoring becomes compromised during exercise or elevated bodily movement due to optical sensor interference [14,32].

These performance constraints engender diminished user confidence and potentially adverse psychological sequelae. Patients encountering false-positive alerts experience heightened health anxiety, reduced perceived well-being, and compromised adherence to prescribed monitoring protocols [79,80]. Clinicians similarly harbor reservations regarding unvalidated wearable data, often preferring verification through conventional electrocardiographic equipment before clinical action, thereby limiting real-world integration into diagnostic workflows [79]. Despite advances in deep-learning-based filtering, the trade-off between sensitivity and specificity remains a clinical and ethical dilemma that complicates the integration of wearable data into diagnostic workflows.

A significant barrier to the widespread clinical integration of wearable cardiac monitoring is the lack of uniform regulatory oversight and standardization. Many consumer-grade smartwatches on the market are not formally classified as medical devices and therefore are exempt from the rigorous validation procedures mandated by authorities such as the FDA or through the European CE mark [81]. This regulatory gap results in considerable heterogeneity regarding data quality, algorithmic transparency, and interoperability between devices, undermining both routine clinical use and large-scale implementation [82]. Recommendations from scientific societies—such as the European Heart Rhythm Association—and technical frameworks like CEN-ISO/TS 82304-2 call for harmonized methods of evaluation and standardized product labeling [15,82,83]. However, adoption and enforcement of these guidelines remain inconsistent, impeding objective comparison of device accuracy or reliability across vendors. In practice, this absence of unified validation protocols leads to methodological weaknesses in device comparison studies and introduces uncertainties into clinical decision-making.

Finally, data privacy and ethical concerns surrounding continuous AF monitoring remain contentious. Wearable devices collect sensitive physiological and behavioral data, often shared with third-party cloud platforms for analytics, raising compliance challenges under the EU’s General Data Protection Regulation (GDPR) [15,55]. Ambiguities in data ownership, informed consent, and secondary use create vulnerabilities for misuse or unauthorized access. Moreover, continuous passive monitoring blurs lines between medical surveillance and personal autonomy, prompting calls for “privacy-by-design” frameworks that embed encryption, anonymization, and consent management within device ecosystems [15,55]. Promoting transparency in data governance and establishing clear internationally harmonized regulatory frameworks for digital health data stewardship are central to fostering ethical trust in wearable-based cardiac care. Collectively, these challenges illustrate that while smartwatch-based AF monitoring holds transformative potential, its safe and equitable integration into clinical practice demands robust technical validation, user inclusivity, and ethical oversight grounded in international regulatory cooperation.

## 8. Clinical Integration and Future Perspectives

The incorporation of smartwatch-derived data into clinical workflows for AF management is advancing rapidly, with mounting evidence for several pivotal applications. Wearable sensors—including PPG and intermittent ECG tracings—generate rich longitudinal data that can be linked with electronic health records, enabling real-time rhythm detection, symptom association, and ongoing therapy surveillance [55]. These continuous streams of information support estimation of AF burden, which has become increasingly central to risk stratification and to guiding decisions regarding anticoagulation therapy or invasive rhythm control strategies [10]. Consensus documents and risk models now frequently call for repeated rhythm monitoring in populations at risk, acknowledging the value of smartwatch algorithms in capturing both symptomatic and clinically silent AF episodes [15,81].

Experience from other cardiovascular conditions illustrates how remote digital technologies can be embedded into structured care pathways and deliver clinically meaningful benefits. In HF management, remote patient monitoring programs that combine telemetric physiological measurements, symptom reporting and protocolized follow-up have been associated with fewer HF hospitalizations and improved quality of life in both randomized trials and real-world studies [84]. A recent meta-analysis of 41 studies encompassing 16,312 patients reported that remote patient monitoring significantly reduced all-cause mortality (pooled OR = 0.81, 95%CI: 0.69–0.95) and first HF hospitalization (pooled OR = 0.78, 95%CI: 0.70–0.87) compared with usual care, particularly when self- management and education components were incorporated [85]. Remote monitoring of cardiac implantable electronic devices has similarly evolved from a technological curiosity to a professional-society-endorsed standard of care: automatic, continuous transmission of device diagnostics now enables earlier detection of arrhythmias, device malfunction and HF decompensation, while safely reducing routine in-person follow-up visits and clinic workload [86]. Telemonitoring reviews further highlight HF and AF as leading use cases for app- and wearable-based cardiovascular monitoring in real-world settings, using combinations of smartphones, smartwatches and connected home devices [87]. Collectively, these precedents support implementing smartwatch-based AF monitoring not as a stand-alone consumer gadget, but as one component within structured, multidisciplinary care pathways.

Looking ahead, the AF care paradigm is poised to be transformed by A and predictive modelling [19]. Machine learning frameworks can integrate extended wearable-derived time series with clinical variables to predict disease evolution, allowing clinicians to identify patients at high risk of complications such as stroke or HF [19]. Such predictive tools promote a shift from reactive management to proactive, individualized care, optimizing the initiation and timing of interventions [20]. Furthermore, the widespread adoption and scalability of wearable monitoring may drive cost-efficiency by reducing unnecessary clinic visits, shortening diagnostic pathways, and minimizing hospitalizations [55,63]. As these systems mature, rigorous clinical validation and seamless integration of wearable-derived metrics remain critical to build clinician trust and to fully harness their potential.

## 9. Conclusions

Recent advances in wearable and AI-driven technologies are transforming AF monitoring by enabling continuous, user-friendly, and highly sensitive detection in daily life. These innovations bridge gaps left by traditional tools, facilitating earlier diagnosis, longitudinal AF burden assessment, and more tailored interventions. Integrating wearables with clinical workflows and leveraging predictive analytics is expected to improve outcomes, patient engagement, and cost-effectiveness. As validation and adoption grow, these technologies promise to reshape AF management, supporting more proactive and individualized care at scale.

## Figures and Tables

**Table 1:** Current studies of wearables in detection of AF recurrence.

First Author, Year	Study Type	Population and Setting	Device/Modality and Time Characteristics	Reference Standard and Time Characteristic	Follow-Up (Months)	Main Outcomes	Clinical Implication
Goldenthal, 2019 [42]	RCT [single center (USA)]	Post-ablation or post DCCV AF patients	AliveCor KardiaMobile, single-lead handheld ECG device daily and when symptomatic	EHR-documented data by clinical ECG source	6	Earlier detection of recurrent AF/AFL vs. control (HR: 1.56, CI 1.06–2.30, *p* = 0.024)	Simple daily mobile-ECG for earlier recognition of AF recurrences
Hermans, 2021 [43]	Prospective within-subject paired comparison	Post-ablation AF patients (paroxysmal)	AliveCor KardiaMobile, single-lead handheld ECG device, 3 recordings/day and when symptomatic	Simultaneous ambulatory ≥24 h Holter ECG at 3,6 and 12 months	12	More AF recurrences (25.2% vs. 14.8%, *p* < 0.001, Se = 95.3%, Sp = 97.5%, PPV = 76.5%, NPV = 99.6%)	Single-lead handheld ECG device as a credible tool for post-ablation follow-up
Huang, 2021 [44]	RCT [single center (China)]	Post-ablation AF patients	BigThumb handheld single-lead ECG, ≥3 recordings/day and when symptomatic	Holter monitoring at 3,6 and 12 months, ECGs if symptomatic	12	More AF recurrences detected (AF-free survival 64.2% vs. 78.9%, *p* = 0.0016), AI algorithm had higher diagnostic accuracy (Se = 94.4%, Sp = 98.5%)	High-frequency handheld ECG improves recurrence detection and influences management (OAC)
Machino, 2022 [45]	Prospective observational, pilot study (Japan)	Post-ablation AF patients	Bra-type device with the dry textile electrode (hitoe) for 3 h the day after ablation	Holter for 3 h the day after ablation(simultaneously)	Single session	Consistency between the two methods, noise count higher in wearable	Comparable performance, noise deemed clinically negligible
Sikorska, 2022 [46]	Prospective within-subject (paired) comparison (AGNES-ECG) [single center (Poland)]	Post-ablation AF patients	Trans-telephonic 6-channel ECG monitoring (HR-2000 recorder), daily and when symptomatic	Holter monitoring at 3,6 and 12 months	12	More AF recurrences documented (24% vs. 14%, *p* = 0.0416) and shorter time-to-first recurrence (156 ± 91 vs. 204 ± 121 days)	Daily intermittent mobile-ECG is a practical, patient-friendly alternative for post-ablation follow up
Senoo, 2023 [47]	Prospective multicenter observational study	Post-ablation AF patients	“Complete” device (ECG and blood pressure monitor) daily	24 h Holter and 12-lead ECG every 3 months	12	More AF recurrences documented (33% vs. 9%) and shorter time-to-first recurrence (40.9 ± 73.9 days faster), benefit greatest with high adherence.	Better and faster detection of AF episodes, adherence matters for diagnostic yield.
Manninger, 2023 [48]	Prospective cohort [single center (Austria)]	Post-ablation AF patients from TeleCheck-AF	Smartphone PPG app (FibriCheck), 3 recordings/day and when symptomatic in the first week after ablation	N/A	18	PPG-suggestive AF in week 1 predicted late ECG-documented recurrence (Se = 65.4%, Sp = 83%, PPV = 89.7%, NPV = 71.9% *p* < 0.001), remote data often triggered interventions	Early PPG monitoring gives actionable prognostic information in the blanking period
Noujaim, 2023 [49]	Post hoc prognostic cohort analysis nested within a multicenter RCT	Post-ablation persistent AF patients from the DECAAF II trial	Handheld smartphone single-lead ECG device (ECG Check, Cardiac Designs), daily in the blanking period	N/A	18	Smartphone ECG-recorded AF burden in the blanking period predicted late recurrence (HR:1.41;95% CI:1.36–1.47; *p* < 0.001)	Quantifying early smartphone ECG-recorded AF burden risk-stratifies patients for later recurrence after ablation
Adasuriya, 2024 [50]	Prospective within-subject (paired) comparison (REMOTE-AF) [dual center (UK)]	Post-ablation long standing persistent AF patients (from CASA-AF)	Consumer wearable (Fitbit) PPG-derived, continuous, 1 min averages in 30 min windows	ILR (Reveal LINQ) or dual-chamber pacemaker, continuous with EGM confirmation	10	Wearable HR ≥110 BPM vs. ILR recurrence: Se = 95.3%, Sp = 54.1%, PPV = 15.8%, NPV = 99.2%	Simple HR-based wearable signals for ruling out recurrence but generate false positives
Sandgren, 2024 [51]	Retrospective sub-analysis of TeleCheck-AF [multicenter (Europe)]	Post-ablation AF patients from TeleCheck-AF	Smartphone PPG app (FibriCheck)with simultaneous symptom logging, 3 recordings/day and when symptomatic	N/A	12	Strong internal correlations for %AF recordings /load/% days (rs 0.88–0.95) and for %symptom recordings/load/%days (rs 0.95–0.98), feasible, high adherence	On-demand PPG can approximate AF burden and patient-reported outcomes
Aguilar, 2025 [52]	Sub-analysis of a multicenter, open-label, RCT [eight centers in Canada]	Post-ablation AF patients in the CIRCA-DOSE trial	Wearable smartwatches (AppleWatch, Fitbit), daytime wear (8:00 AM–10:00 PM)	ILR (Reveal LINQ), continuous monitoring	12	Wearable smartwatch: high sensitivity (highest 82%), estimated AF burden strongly correlated with AF burden from ILR (r > 0.97) and AF burden in missed cases was low (<0.2%)	Smartwatches can be used as a follow-up method in post-ablation patients

## Data Availability

Our study data are available from the corresponding study author (PS) upon reasonable request.

## Abbreviations

The following abbreviations are used in this manuscript:

AF	Atrial Fibrillation
AFL	Atrial Flutter
AI	Artificial Intelligence
AFEQT	Atrial Fibrillation Effect on QualiTy of Life
BPM	Beats Per Minute
CI	Confidence Interval
CNN	Convolutional Neural Networks
DCCV	Direct Current Cardioversion
ECG	Electrocardiography
HER	Electronic Health Records
GDPR	General Data Protection Regulation
HF	Heart Failure
HR	Hazard Ratio
IHRN	Irregular Heart Rhythm Notification
ILR	Implantable Loop Recorders
LED	Light Emitting Diodes
NPV	Negative Prognostic Value
OAC	Oral Anticoagulation
OR	Odds Ratio
PACs	Premature Atrial Contractions
PPG	Photoplethysmography
PPV	Positive Prognostic Value
PVCs	Premature Ventricular Contractions
RCT	Randomized Clinical Trial
Se	Sensitivity
Sp	Specificity
